# Editorial: Endocrine-Disrupting Compounds in Plastics and Their Effects on Reproduction, Fertility, and Development

**DOI:** 10.3389/ftox.2022.886628

**Published:** 2022-03-25

**Authors:** Francesca Maradonna, Laura N. Vandenberg, Rosaria Meccariello

**Affiliations:** ^1^ Department of Life and Environmental Sciences, Polytechnic University of Marche, Ancona, Italy; ^2^ School of Public Health and Health Sciences, University of Massachusetts – Amherst, Amherst, MA, United States; ^3^ Department of Movement Sciences and Wellness, University of Naples Parthenope, Naples, Italy

**Keywords:** endocrine disruptors, plastics, reproduction, fertility, development

One of the main consequences of industrialization is the development and daily use of plastics, including numerous related additives and contaminants (of emerging concern) that are released in the environment. Many of these chemicals bio-accumulate in biological tissues and can adversely affect human and wildlife health by altering endocrine functions or through other biological mechanisms (Zoeller et al., 2016). Therefore, living organisms, including humans, are inevitably exposed to endocrine disrupting chemicals (EDCs) by dermal contact, ingestion or inhalation of these ubiquitously distributed environmental toxicants.

Numerous EDCs have been shown to exert long-term pleiotropic effects on endocrine function and health, and contribute to endocrine-mediated diseases (Gore et al., 2015; La et al., 2020). Compounds that are commonly used for the production of daily use goods including food and drink packaging like bisphenol A (BPA) and its analogues (BPS, BPF, BPB, BPAF), phthalates like bis-(2-ethylhexyl)-phthalate (DEHP) and dibutyl-phthalate (DBP), and per- and poly-fluoroalkyl substances (PFAS, like PFOS and PFOA), among others, enter into both the environment and living organisms, thus posing ecotoxicological and health risks (Vandenberg, 2021). These risks have been well-acknowledged by scientific and medical experts. For example, a 2020 report from experts working with the Endocrine Society and the International Pollutants Elimination Network provided “clear and extensive evidence of the human health impacts of many chemicals in common plastics” on outcomes including cancer, diabetes, metabolic disorders, thyroid diseases, neurological outcomes, and infertility, among others (Flaws et al., 2020).

An additional urgent and emerging concern is the environmental exposure to plastic debris in the micro and nano-range that enter the food chain. Although the number of studies of micro- and nanoplastics evaluating harmful effects on aquatic and terrestrial organisms remains small, evidence indicates that even remote environments (e.g., the arctic) are contaminated with these pollutants (D'Angelo and Meccariello, 2021). There are several concerns regarding micro- and nanoplastics. First, these plastics leach EDCs, similar to how whole plastic products leach EDCs. Second, microplastics act as molecular sponges, concentrating EDCs at higher concentrations than those usually detected in the environment (Wang et al., 2015; Deng et al., 2021).

Reproduction, fertility and development represent several of the main targets of environmental toxicants, with different outcomes on health depending on dose, timing of exposure, and timing of evaluation (Vandenberg and Turgeon, 2021). Gametogenesis and gamete quality, embryo development and pregnancy, pre- and postnatal developmental processes are highly sensitive to effects of exposures to plastics derived EDCs (Gore et al., 2015). Tissue damage, poor gamete quality, low fertility rate and developmental abnormalities are some of the consequences of developmental exposures to these compounds.

While the effects in the exposed adults are often, but not always, transient, gestation and the early postnatal period are critical exposure windows. Importantly, the effects of exposures during early development may not manifest for years, or even decades (Heindel and Vandenberg, 2015). The effects of reproductive toxicant exposures are not only observed in both exposed subjects but also in subsequent generations through the inheritance of deregulated epigenetic markers from gametes or the establishment of unsuitable epigenetic signature during early embryo development (Chianese et al., 2018; Santoro et al., 2019).

A large body of experimental evidence from studies using animal models and cell lines continues to reveal adverse effects on reproductive and developmental outcomes after exposure to these compounds. Fully characterizing the effects, and understanding the mechanisms by which EDCs induce harm, indicates the need for further studies in the field, as well as the development of safe alternatives to preserve reproduction, fertility and health in humans. In this Research Topic we present a collection of two original research articles and two review articles.

The first research article (Bottalico et al.) utilized a non-mammalian model (the goldfish, *Carassius auratus*) to characterize the effects of BPA, nonylphenol, DEHP and fucosterol - alone or in combination - on metabolomic profiles of midbrain, testis and liver. This seasonal breeder requires specific neuroendocrine signaling to allocate the metabolic resources that sustain growth and reproduction. EDC exposures affected metabolism, but these studies revealed that the mid-recrudescence stage was most vulnerable to metabolic perturbation in male fish. Thus, seasonally driven physiological changes may alter the vulnerability of seasonal breeders to EDCs.

The second research article aimed at characterizing the effects of co-exposures to BPA and hops extracts on adult rat behavioural responses following exposures during adolescence (Morin et al.). This study contributes to evidence that BPA disrupts social behaviors (Patisaul and Belcher, 2017), but provides new evidence for the sensitivity of the pubertal period. Recent work aims to identify impactful ways to counteract BPA toxicity through incorporation of nutraceuticals in the diet. Results obtained suggest that BPA- and hops-exposed rats differed in metrics of anxiety, providing preliminary evidence that hops extracts might counteract BPA neurotoxicity.

In the first review article, the peer-reviewed literature on the reproductive effects of PFAS was summarized (Chambers et al.). Evidence from human populations suggests that PFAS exposures are associated with effects on fertility, fetal growth, pre-eclampsia and pregnancy-induced hypertension, thyroid hormone levels in pregnant women, and risk of pre-term birth. Importantly, PFAS is a large class of chemicals and most of the evidence accumulated to date comes from studies of PFOA and PFOS; this review also examines the effects of short-chain replacements such as GenX, ADONA, and F53B, which have been detected in the environment but have limited studies from experimental models, and little (or no) evidence from human populations.

The final contribution to this Research Topic examines the evidence that microplastics affect reproductive health, using *Caenorhabditis elegans* and other non-vertebrate species as models (Kevei et al.). Although there are relatively few studies examining the effects of exposures to plastic particles, there is some evidence that plastic particles decrease growth rates, reduce fertility and/or fecundity, and induce other measures of toxicity. The effects of nano- and microplastics can differ depending on their shape, size and composition, and they may also aggravate the effects of other pollutants.

There is overwhelming evidence, acknowledged by scientific and medical organizations, that EDCs induce harm to humans and wildlife. Effects on reproduction, development and neurobehaviors are well documented and the manuscripts included in this Research Topic add to that growing but convincing body of evidence.
